# Testosterone variation in a semi-captive population of Asian elephants in Myanmar

**DOI:** 10.1093/conphys/coae076

**Published:** 2024-11-22

**Authors:** Héloïse Moullec, Vérane Berger, Diogo J Santos, Susanna Ukonaho, Lisa Yon, Michael Briga, U Kyaw Nyein, Virpi Lummaa, Sophie Reichert

**Affiliations:** Department of Biology, University of Turku, Vesilinnantie, 5, Turku 20014, Finland; Department of Biology, University of Turku, Vesilinnantie, 5, Turku 20014, Finland; Department of Biology, University of Turku, Vesilinnantie, 5, Turku 20014, Finland; Department of Biology, University of Turku, Vesilinnantie, 5, Turku 20014, Finland; School of Veterinary Medicine & Science, University of Nottingham, Sutton Bonington Campus, Leicestershire, LE12 5RD, United Kingdom; Department of Biology, University of Turku, Vesilinnantie, 5, Turku 20014, Finland; Myanma Timber Enterprise, MONREC, West gyogone, Insein Township, Yangon, Myanmar; Department of Biology, University of Turku, Vesilinnantie, 5, Turku 20014, Finland; Department of Biology, University of Turku, Vesilinnantie, 5, Turku 20014, Finland

**Keywords:** Asian elephant, environmental factors, glucocorticoid, H/L ratio, hormones, oxidative stress, testosterone**Abbreviations:** T: testosterone; FTM: faecal testosterone metabolite; FGM: faecal glucocorticoid metabolite; H/L ratio: heterophils and lymphocytes ratio; ROMs: reactive oxygen metabolites; SOD: superoxide dismutase

## Abstract

Hormones are known to be involved in life-history trade-offs as systemic signals that establish functional links among traits and regulate key behavioural and physiological transitions between states in organisms. Although major functions of many steroid hormones such as testosterone are conserved among vertebrates, circulating concentrations vary widely both within and across species, and the degree to which observed hormone concentrations mediate life-history responses to environmental variation is less understood. In this study, we investigated how faecal testosterone metabolite (FTM) concentrations varied with extrinsic and intrinsic factors. To do so, we took advantage of a 6-year period of longitudinal sampling of FTM, indicators of stress and oxidative status in a semi-captive population of Asian elephants (*n* = 3163 samples from 173 individuals) in Myanmar. We determined how the variation in FTM is associated with age, sex, origin (captive-born or wild-caught), seasonality of the environment, individual stress level [measured by faecal glucocorticoid metabolite (FGM) and heterophil to lymphocyte ratio (H/L)] and oxidative status (reactive oxygen metabolite concentrations and superoxide dismutase activity). We reported that FTM increased with age from juvenile to adulthood for both sexes, with higher FTM concentrations in males than females. Moreover, elephants showed significantly higher FTM concentrations during the hot season and monsoon than in the cold season. However, for the physiological indicators, we found contrasting results. While FTM concentrations were strongly positively correlated with FGM concentrations, FTM concentrations were not related to H/L ratios. Finally, we found no relationship between FTM and the oxidative status of individuals. Our study provides new insights on the factors associated with variation in testosterone concentrations—a key hormone for reproduction and fitness of individuals—in Asian elephants living in their natural environment, which has relevance for effective conservation measures of this endangered species.

## Introduction

Testosterone, a steroid hormone produced by gonads, is central in the development of reproductive organs and spermatogenesis, and secondary sexual characteristics involved in mating and reproduction (Ketterson *et al.,* 1996; Ketterson and Val, 1999; Wingfield *et al.,* 2001). Testosterone supports phenotypic traits that enhance attractiveness and increase mating effort (Wingfield, 1984; Ketterson and Val, 1999; Stoehr and Hill, 2000; McGlothlin *et al.,* 2007; Mills *et al.,* 2009; Negro *et al.,* 2010). However, the secretion of testosterone also induces costs for the organisms. High testosterone concentrations can decrease survival rate (Marler and Moore, 1988; Dufty, 1989; Reed *et al.,* 2006; Mills *et al.,* 2009) and parental care (Silverin, 1980; Hegner and Wingfield, 1987; Oring *et al.,* 1989; Stoehr and Hill, 2000; O’Neal *et al.,* 2008; Veiga *et al.,* 2008) and can negatively impact female reproductive success and fitness as observed in birds (Rutkowska *et al.,* 2005; O’Neal *et al.,* 2008; Veiga *et al.,* 2008). In mammals, an excess of testosterone during prenatal development can cause physiological disruption in adulthood, including disruptions of reproductive processes (Padmanabhan *et al.,* 2006). Therefore, testosterone has a direct influence on the fitness of individuals (Wingfield *et al.,* 2001).

While the role of steroid hormones is conserved among vertebrates, testosterone concentrations vary both between and within individuals (Kempenaers *et al.,* 2008). Males display higher concentrations of testosterone than females in mammals (Rasmussen *et al.,* 1984; Glickman *et al.,* 1987, 1992; Holekamp and Talamantes, 1991; Whitworth *et al.,* 1999; Goymann *et al.,* 2001; Dittami *et al.,* 2008; Burgess *et al.,* 2012), and the concentrations vary according to life stage or lifespan (Kempenaers *et al.,* 2008). In some long-lived mammals, the mean testosterone concentration increases with age, as in male elephants (Brown *et al.,* 2007), but decreases in very old individuals (Thongtip *et al.,* 2008). Similarly, in primates, androgen hormone levels increase with age (Martin *et al.,* 1977), with the highest testosterone concentrations observed at maturity, after which they decline (Beehner *et al.,* 2005, 2009; Seraphin *et al.,* 2008) or reach a plateau (Crawford *et al.,* 1997). However, age-related variation in testosterone concentrations among taxa still needs to be assessed, especially in females, since studies to date have mainly focused on males.

Beyond individual characteristics, testosterone secretion is influenced by extrinsic factors from the environment, sometimes reflecting the adaptive mechanisms of an individual in a changing environment (Wingfield and Kenagy, 1991; Astheimer *et al.,* 1995). Seasonality, marked by variations in climate, food availability, disease prevalence and predation, is an important ecological factor known to affect testosterone concentrations (Whitehead and McEwan, 1973; Pelletier *et al.,* 1982; Orlando *et al.,* 2003; Gündoğan, 2007; Sun *et al.,* 2011). In captive Asian elephants in Thailand, for example, males display seasonal variation in T, with higher testosterone concentrations during the cold season and monsoon, when food availability is higher than in the hot season (Thongtip *et al.,* 2008; Norkaew *et al.,* 2019). Hence, environmental variation can affect hormonal concentrations, and it is essential to consider this in the study of testosterone concentration variation, as intrinsic factors can be confounded with environmental effects (Kempenaers *et al.,* 2008).

Some environmental fluctuations (e.g. climate, food availability) cause an activation of the hypothalamic–pituitary–adrenal (HPA) axis with the production of glucocorticoids, resulting in the suppression of the hypothalamic–pituitary–gonadal (HPG) axis, and thus lower testosterone levels. This decrease in testosterone secretion following a stress event has been evidenced in male vertebrates, such as humans (Matsumoto *et al.,* 1970; Kreuz *et al.,* 1972; Aakvaag *et al.,* 1978), tree lizards (Moore *et al.,* 1991), rats (Gray et al., 1978) and silver foxes (Moe and Bakken, 1996), and could disrupt or suppress reproduction (Sapolsky, 2002; Wingfield and Sapolsky, 2003). Concentrations of glucocorticoids, including corticosterone, are often used as an indicator of stress in various mammalian species (Keay *et al.,* 2006). Similarly, the ratio between two types of white blood cells—heterophils (or neutrophils in most mammals) and lymphocytes—referred to as heterophil to lymphocyte ratio (H/L) ratio, is considered a fairly reliable stress marker, with decreasing lymphocytes in blood and increasing neutrophils or heterophils in response to stress (Gross and Siegel, 1983; McFarlane and Curtis, 1989; Vleck *et al.,* 2000). These two physiological responses to a stressor are correlated (Seltmann *et al.,* 2020), but they do not have the same role and differ temporally in their response to stress (Davis and Maney, 2018). They might offer ways of elucidating the interplay between endocrinological and haematological responses, and how they might interact in phenotypic plasticity.

Another physiological stress consequence is oxidative stress, manifested by overproduction and accumulation of free radicals as a result of an imbalance between the production of reactive oxygen species (ROS) and the activation of various antioxidant defences (Halliwell, 1994). Previous studies, for example, on rats (Chainy *et al.,* 1997; Pansarasa *et al.,* 2002), rabbits (Aydilek *et al.,* 2004), humans (Zhu *et al.,* 1997) and birds (Alonso-Alvarez *et al.,* 2007), showed that oxidative stress increased concomitantly with increasing concentrations of testosterone. Testosterone can increase ROS production and impair the antioxidant system through genomic and non-genomic effects (see for example Chainy *et al.,* 1997; Cruz-Topete *et al.,* 2020). However, other studies have obtained different or even contradictory results. In a study on White’s skinks, Isaksson *et al.* (2011) found that an aggressive behavioural phenotype, rather than testosterone concentrations, was associated with higher oxidative stress. Furthermore, some medical studies found a protective effect of testosterone reducing oxidative stress in the brain (Túnez *et al.,* 2007; Meydan *et al.,* 2010; Toro-Urrego *et al.,* 2016; Yan *et al.,* 2017; Cai and Li, 2020). Hence, further studies on a wider range of species investigating the link between testosterone variation and the oxidative status of individuals are needed to better understand the influence of testosterone on the fitness of individuals.

Although there is extensive research on the role of testosterone, we know far less about the determinants of its variation and the mechanisms mediating the hormonal response to environmental changes. In this study, we investigated the intrinsic and extrinsic determinants of faecal testosterone metabolite (FTM) variation in the Asian elephant measured from faecal samples, a non-invasive method used in numerous studies including on Asian elephants (Seltmann *et al.,* 2020; de Andrés *et al.,* 2021; Glaeser *et al.,* 2021; LaDue *et al.,* 2022, 2023; Ukonaho *et al.,* 2023). Previous studies on the testosterone endocrinology of the Asian elephant focused primarily on males during the rutting period called musth, characterized by a peak of testosterone concentrations, high aggressiveness and high sexual activity (Rasmussen and Perrin, 1999; Brown *et al.,* 2007; Yon *et al.,* 2007a, 2007b, 2008; Kumar *et al.,* 2014). In contrast, very few studies have examined testosterone variation at a basal concentration in both sexes. We took advantage of our unique dataset on a semi-captive population of elephants in Myanmar, working during the day in timber camps managed by the Myanma Timber Enterprise (MTE), but free roaming at night in the surrounding forest. We collected 3163 FTM samples from 173 elephants over 6 years and analysed the association between FTM variation and individual characteristics (age, sex, wild-caught or captive-born origin), the environment (seasonal effect) and potential indicators of physiological stress [faecal glucocorticoid metabolite (FGM) concentrations and H/L ratio] and the possible consequences of FTM variation on the oxidative status of individuals [reactive oxygen metabolite (ROM) concentrations and superoxide dismutase (SOD) activity]. Determining the link between ecological and physiological factors with testosterone level, a crucial hormone for reproduction and fitness, is important to understanding the survival and reproduction patterns of the Asian elephant. By identifying the factors affecting testosterone variation in Asian elephants, our study will provide essential information to develop targeted conservation strategies and may offer management solutions for this endangered species. We expected the following: (1) higher FTM in males and (2) sexually mature individuals, but lower in the oldest elephants; (3) a seasonal effect, known to affect health, markers of stress and mortality risks in our population (Mumby *et al.,* 2013a, 2013b; dos Santos *et al.,* 2020; Ukonaho *et al.,* 2023), with higher FTM concentrations during monsoon due to higher food availability, which we expect would lead to a greater activity of the HPG axis. For the underlying physiological factors, we hypothesized (4) a suppressive effect of stress on FTM, thus a negative relationship between FGM concentrations and H/L ratios with FTM concentrations. We also predicted an origin-related variation in FTM concentrations, with lower concentrations for wild-caught than captive-born elephants as a result of the higher stress levels they experience in captivity (Lahdenperä *et al.,* 2018; Jackson *et al.,* 2019). Finally, we expected (5) a negative effect of higher FTM concentrations on the oxidative status of individuals, i.e. elevated biomarkers of oxidative stress.

## Materials and Methods

### Study model

We studied 173 semi-captive elephants from Myanmar, owned by the governmental MTE and employed in the timber industry (Schmidt and Mar, 1996). These elephants were managed during the day by their allocated handler (called a mahout) in logging camps to handle and pull logs, but they were free to roam at night and outside of working hours (5 to 8 hours during the day) in the surrounding forests. Most of these timber elephants were captive-born, but some had been captured from the wild. Since 1968, the capture of wild elephants was restricted to elephants of at least ~5 years of age in Myanmar, and it was banned more than two decades ago (Schmidt and Mar, 1996; Lahdenperä *et al.,* 2018). Asian elephants can reproduce all year round, as female ovulation and musth in males can occur in any season (Fowler and Mikota, 2006; Brown *et al.,* 2007; Hildebrandt *et al.,* 2011). Reproduction in logging elephants is not managed by humans, and they can breed with wild elephants when released into the forest. Hence, these MTE elephants exhibit many foraging and breeding patterns comparable to those of wild elephants and are termed ‘semi-captive’ (Robinson *et al.,* 2012; Lahdenperä *et al.,* 2018).

Each of these semi-captive elephants was marked with a unique identification (ID) number and was regularly checked from birth to death by an MTE veterinarian, who recorded a wide range of life-history information in the elephant’s logbook, such as their origin (captive-born or wild-caught), health status, morphological data, pedigree (mother ID) and dates of birth and death. The exact dates of birth of wild-caught elephants were estimated, as they were unknown, in contrast to captive-born elephants, for whom the specific day, month and year of birth was known. The approximate age of wild-caught elephants was determined by comparing their body height and mass to those of captive-born elephants of known age (Lahdenperä *et al.,* 2018; Lalande *et al.,* 2022). In any given year, elephants in Myanmar experience three distinct seasons: cold season (October–January), hot season (February–May) and monsoon (June–September). Traditionally, elephants work during monsoon and cold seasons and rest during the hot season (Mumby *et al.,* 2013a, 2015; Lahdenperä *et al.,* 2018), but none of the elephants included in the current study were involved in heavy work at time of the sampling.

### Determination of age classes

The population of MTE elephants has a predicted median lifespan of 30.81 years for males and 44.73 years for females (Lahdenperä *et al.,* 2018). The age of the elephants in the current study ranged from a few months to 69 years old for the females, and from a few months to 62 years old for the males. We created five different age classes for the elephants, which followed both the life history and the work life of the general population. These classes were calves-at-heel (0–4 years old), weaned calves (5–10 years old), adolescents (11–20 years old), prime-aged elephants (21–54 years old) and elderly elephants (55+ years old). MTE elephant calves stay with their mother from birth to 4–5 years old when they are weaned. The interbirth interval is around 4–5 years in Asian elephants (Sukumar, 2003; Lahdenperä *et al.,* 2014). At 5 years old, calves are separated from their mother to be tamed (Crawley *et al.,* 2020). The taming process lasts around 4 weeks, at the end of which each calf is assigned to an individual handler and continues to be trained (5–10 years old) without working duties yet. Between 11 and 20 years old, adolescent elephants conduct light work only. They usually reach sexual maturity at around 14–15 years. Prime-aged elephants aged 21–54 years old are all adults. The highest fertility of female elephants is between 20 and 50 years old and declines after 50 years old, with a peak in births occurring when females are aged 20–25 years old (Lahdenperä *et al.,* 2014). These prime-aged elephants belong to the workforce, when they drag heavier logs during full-time work, before they retire from work at 55 years old. The workloads are imposed and regulated for all elephants by MTE (Mar *et al.,* 2012).

### Faecal sample collection, testosterone metabolite extraction and hormone assays

#### Collection and extraction

Our study was conducted in Kawlin logging camps, in Sagaing region of Myanmar. It includes 173 elephants longitudinally sampled for faecal samples once a month (when possible) across 6 years, from 2013 to 2018, corresponding to an average of 56.3 ± 10 faecal samples collected monthly and used in this study. These samples were collected just after defecation in the morning, generally between 6 a.m. and 9 a.m., by local veterinarians as part of their regular health monitoring. For male elephants, the sample collection was conducted for safety reasons outside musth, a period of high sexual activity characterized by a peak of testosterone and glucocorticoid hormones, and high aggressiveness. An aliquot of exactly 4.5 g was taken from each faecal bolus. The samples were transferred to a cooler box for a maximum of 8 hours before being stored in a freezer at −20°C.

Previously validated faecal hormone assays (Watson *et al.,* 2013; Kumar *et al.,* 2014) were used to measure FTM and FGM concentrations. Faecal samples were analysed in 12 separate batches at the Veterinary Diagnostic Laboratory, Chiang Mai University, Thailand. The faecal samples were dried in a hot air oven at 50°C before extracting the testosterone metabolites using a boiling extraction method (Crawley *et al.,* 2021; Supanta *et al.,* 2024). Samples (~0.1 g) were placed in glass tubes with 5 ml of 90% ethanol and extracted by boiling in a water bath for 20 minutes, with 100% ethanol added to maintain the volume at 5 ml. Samples were then centrifuged, the faecal supernatants were combined and dried in a 50°C water bath, reconstituted by vortexing for 1 minute in 3 ml of ethanol, dried down again and finally re-suspended in 1 ml of methanol. Samples were diluted 1:3 in assay buffer and stored at −20°C until analysis. We then quantified the concentrations of testosterone metabolites by enzyme immunoassay (EIA) validated for elephants (Kumar *et al.,* 2014, see below for details), using a rabbit anti-testosterone polyclonal antibody (R156/7) with a concentration of 1:110 000 and a horseradish peroxidase-conjugated testosterone tracer (HRP) with a concentration of 1:20 000. The sample dilution of the faecal samples was 1:4.

The FTM data used in this study consisted of 3163 observations from 173 individuals including 106 females and 67 males (1 to 54 observations per individual with an average of 18). FTM concentrations ranged from 6.04 to 281.4 ng/g of dry faeces (with outliers removed, see Statistical Analyses section), and the age of sampled individuals represented a wide range from a few months old to 69 years old.

#### Plate coating with secondary antibody

96-well plates were pre-coated with 150 μl (10 μg/ml) of coating antibody (goat anti-rabbit IgG) diluted in coating buffer (16 mM Na_2_HPO_4_, 1.6 mM NaH_2_PO_4_; pH 8.0), covered with a plate cover and then stacked and left for 24 hours at ambient temperature. The coating buffer was then removed from each plate, and 0.25 ml of blocking buffer (0.1% Tween 20, 8.12 mM sodium phosphate dibasic, 1.88 mM sodium phosphate monobasic, 15 mM sodium chloride, 0.009% Kathon CG/ICP, 29.21 mM sucrose) was dispensed into each well. Blocked plates were stacked and left for 4–24 hours at room temperature. The wells were then emptied, blotted dry, and the plates were transferred to a desiccator and left at ambient temperature until the inside humidity reached less than 20%. The dried plates were then packaged individually and heat sealed inside labelled foil Ziploc bags before being stored at 4°C until used for EIAs.

#### Enzyme immunoassay

The pre-coated goat anti-rabbit plates were used. Seventy-five microliters of assay buffer (0.1% Tween 20, 13.68 mM Trizma base, 86.3 mM Trizma hydrochloride, 0.15 M sodium chloride, 10 mM ethylenediaminetetraacetic acid (EDTA), 0.09% Kathon CG/ICP, 0.1% bovine serum albumin (BSA), Standard Grade pH 7.0) was added to the non-specific binding (NSB) wells, and 50 μl of assay buffer were added into the other wells to act as maximum binding wells (Bo or 0 pg/ml). Then, 50 μl of testosterone standards (17-hydroxy-4-androsten-3-one, Steraloids Cat. #A6950; range from 0.047 to 12 ng/ml), quality controls, and samples were each added into a well in the plate. Twenty-five microliters of HRP with a concentration of 1:20 000 was immediately added to each well, followed by 25 μl of testosterone polyclonal antibody (R156/7) with a concentration of 1:110 000 added to all wells except the NSB wells. The plate was then covered with a plate sealer and shaken at ambient temperature for 2 hours. Each well was washed four times with wash buffer (1:20 dilution), and 100 μl of 3,3',5,5'-Tetramethylbenzidine (TMB) substrate solution (KPL TMB Microwell Peroxidase Substrate System 2-contents) was added to each well before incubation at ambient temperature for around 30 minutes without shaking. Finally, 50 μl of stop solution (2 M H_2_SO_4_) was added to each well, and the optical density generated from each well was read at 450 nm by a microplate reader. The assay had a sensitivity of 0.0567 ng/ml, with intra-assay coefficients of variation (CVs) lower than 10% and inter-assay CV of 12.73%. The assay sensitivity refers to the lowest concentration that can be accurately analysed, which corresponds to the lowest standard concentration, calculated in Magellan Software using a 4PL curve.

### FGM extraction and hormone assay

FGM concentrations were measured by EIA using an assay validated for use in Asian elephants (see above for plate coating and EIA protocol). The plate was filled with samples and corticosterone standards (Sigma, 27 840; 50 μl; range from 0.078 to 20 ng/ml), followed by corticosterone-HRP (25 μl; 1:30 000) and polyclonal rabbit anti-corticosterone antibody (CJM006, Coralie Munro, UC Davis, CA) (25 μl; 1:100 000), and each plate was shaken at ambient temperature for 1 hour. Following Watson *et al.* (2013) protocol, we utilized Nunc MaxiSorp® plates, with substrate reagents at ambient temperature, and dark incubation conditions to optimize the results. The intra- and inter-assay CVs were lower than 10% and equal to 11.8%, respectively. The assay sensitivity was 0.099 ng/ml, and the absorbance was measured at 450 nm. Data consisted of 2964 observations from 171 individuals (1 to 51 observations per individual with an average of 17) ranging in age from 0 to 69 years old. Glucocorticoid metabolite concentrations ranged from 3.2 to 409.7 ng/g of dry faeces (similar to de Andrés *et al.,* 2021).

### Blood samples collection

Blood samples were collected once per season (three times/year), corresponding to an average of 39.9 ± 19.6 blood samples collected per season and used in this study. Blood was collected from an ear vein of each elephant using a Vacuette® system (Greiner Bio-One, Kremsmünster, Austria) in the morning of non-workdays by local veterinarians as part of their routine health monitoring of each elephant for which they have been trained. Blood samples were collected into 8-ml tubes with the anticoagulant EDTA for measuring the H/L ratio, and in 9-ml serum separator tubes for oxidative stress measurement (dos Santos *et al.,* 2020). The H/L ratio was measured the same day, within 0 to 8 hours after collection (see Methods below). The serum separator tubes were centrifuged (RCF—1320 g) for 20 minutes 3 to 6 hours after collection. Around 2 ml of serum was then pipetted into separate tubes and frozen at −20°C. The serum samples were later transported to the University of Turku in Finland and stored at −80°C until used for oxidative stress measurement.

Blood samples were collected less frequently than faecal samples, resulting in larger numbers of samples for the faecal hormone analyses and smaller sample numbers for the measurements of H/L ratio and oxidative stress. All sample sizes are summarized in Table A1 in the supplementary materials.

### Evaluation of H/L ratio

From blood samples collected three times in 2016, 2017 and 2018 during the three seasons (hot, cold and monsoon) in Myanmar, we assessed the ratio between heterophils and lymphocytes (H/L), another indicator of stress and arousal, which correlates with glucocorticoid concentrations in elephants (Seltmann *et al.,* 2020). Seltmann *et al.* (2020) recommend including behavioural indicators of stress, which we do not have in this study, to help interpret physiological markers of stress. To measure the H/L ratio, we used a validated protocol for Asian elephants (Seltmann *et al.,* 2020). We kept the blood samples in 8-ml tubes containing the anticoagulant EDTA (dos Santos *et al.,* 2020). We stained blood samples collected in EDTA with Romanowsky stain and manually counted the number of leucocytes from the blood smears. Then, we calculated the ratio of heterophils to lymphocytes counted on each blood smear. H/L ratios from the individuals used in this study ranged from 0.21 to 5.6. These data consist of 274 observations from 89 individuals (1 to 6 observations per individual with an average of 3), ranging in age from 4 to 69 years old.

### Oxidative stress assessment

To investigate the physiological consequences of FTM variation, we also measured markers of oxidative status of the elephants from blood samples collected from 2014 to 2018 in Myanmar. Two markers of oxidative status were evaluated in plasma: ROMs, indicators of oxidative damage, and the activity of SOD, indicative of antioxidant defence. These analyses were performed at the University of Turku, Finland.

#### Reactive oxygen metabolites

We measured the concentration of ROMs using a d-ROMs Test kit (Diacron International) according to methods reported by Costantini and Dell’Omo (2006). The d-ROMs test measures hydroperoxides (ROOH), the main compounds contributing to the oxidant ability of the plasma (Costantini, 2016). Each well of a 96-well microplate (SpectraPlateTM; PerkinElmer®) was filled with 5 μl of serum samples and 200 μl of “master mix” from the d-ROMs Test kit. The plates were shaken for 10 seconds and incubated at 37°C for 75 minutes before being read with a spectrophotometer (PerkinElmer®) at 510 nm. ROMs concentration is expressed as mg H_2_O_2_ equivalent/dl. The concentration of ROM measured in individuals in this study ranged from 4 to 30.5 H_2_O_2_/dl, and the intra-individual variation based on duplicates was 5.12 ± 1.02%. These data consist of 356 observations from 81 individuals (1 to 11 observations per individual with an average of 4), ranging in age from 4 to 64 years old.

#### Superoxide dismutase

SOD is an antioxidant enzyme involved in the first step of the cascade catalysing the dismutation of superoxide anion free radical (O_2_^−^) into oxygen and hydrogen peroxide; this reduces the amount of O_2_^−^, which can damage cells at high concentrations. We measured SOD enzymatic activity from 25 μl of serum samples using a SOD activity kit (Enzo® Life Sciences, USA) following the manufacturer’s instructions. The plates were read at 450 nm with a spectrophotometer (PerkinElmer®). This test quantifies *in vitro* the kinetics of inhibition in superoxide formation resulting from SOD antioxidant activity. SOD activity from the individuals used in this study ranged from 15.4 to 683.9 U/ml (units of enzymatic activity per milliliter), and the intra-individual variation based on duplicates was 23.3 ± 4.64%. These data consist of 438 observations from 91 individuals (1 to 13 observations per individual with an average of 5), ranging in age from 4 to 65 years old.

### Statistical analyses

We investigated the association of season, individual characteristics (age, sex, origin) and indicators of stress (FGM, H:L ratio, oxidative status) with FTM concentrations. All analyses were carried out on R software version 3.6.3 (R Core Team, 2022), and the graphical representations were constructed with ‘ggplot2’ package (Wickham, 2016). These figures show on the Y axis the values of FTM concentrations, in function of an explanatory variable, corrected for all covariates in the model. Correcting for covariates is a method well supported by other studies (e.g. Briga *et al.,* 2019; Briga and Verhulst, 2021) to provide a figure that matches the tested model, and because covariates are accounted for, it prevents showing false positives or false negatives.

First, we analysed the distribution of FTM concentrations measured and the presence of potential outlier data points using *horn.outlier* function (package ‘referenceIntervals’; Finnegan, 2015), which identifies data points outside 1.5*IQR (intequartile range) from the first or third quartile as an outlier.

We detected 48 outliers from 3211 observations (1.49% of all data points), which were removed from our analysis. The final dataset contained 3163 observations from 173 elephants. We applied a logarithmic transformation to the FTM values, to fit them to a normal distribution, before using linear mixed models (package ‘lme4’, Bates *et al.,* 2015), adjusted with the maximum likelihood method. We used the summary of the model to obtain the model parameters and *P* value of each variable. To compare the significance between the modalities of the factors in these models, we performed *post hoc* tests with the function ‘difflsmeans’ from the package ‘lmerTest’ (Kuznetsova *et al.,* 2020). This function carries out a Satterthwaite approximation for degrees of freedom.

First, we built a ‘full model’ in which we tested the effect of season and intrinsic variables on FTM concentrations. This model included five fixed factors: sex, age, class (calves-at-heel, weaned calves, adolescents, prime-aged elephants or elderly elephants), elephant origin (captive-born or wild-caught) and season (hot, cold or monsoon). To account for repeated measures on the same individuals, we included the ID of the elephants as a random factor. We also accounted for temporal and laboratory variation by including the year of sample collection and batch as random factors. When we faced singularity issues in the models, signalled by a warning from R, we removed the random factor(s) (year or batch) that had a variance near zero. In this first model, we also tested the interactions between the sex and age class of the elephants and between the sex and the season of the elephants to investigate whether FTM concentrations varied by age, and/or differently in males and females, and whether environmental conditions affected the FTM concentrations of males more than that of females.

From the first ‘full model’, we then determined a ‘best model’ to test the effect of FGM and H/L ratio. We selected this model using the second-order Akaike information criterion (AICc) given with the function ‘dredge’ of ‘MuMIN’ package (Burnham *et al.,* 2011; Barton, 2022). This best model kept only the variables explaining variation in FTM concentrations: the age class, sex and season, and the same random factors as the ‘full model’: elephants’ ID, year, batch (see Table A6 in the appendix). To test the effect of stress variables, FGM and HL ratio, on FTM concentration variation, we separately added to the ‘best model’ one of these stress indicator variables at a time to determine its influence. We had to include these variables in separate models as the H/L ratio was available for a smaller sample of animals and data points than FGM, since faecal samples were collected monthly versus once per season for blood samples. Thus, separate models were developed because including both variables in the same model would have unnecessarily limited the sample size of FGM.

**Table 1:** Output of the linear mixed model, testing the effect of the origin of the elephants (captive-born or wild-caught), sex, age class and season on FTM concentrations, obtained with the summary of the model. As the interactions were not significant, the model was run again without the interactions, and we indicated only the *P* value given by the Type III analysis of variance. Values in bold indicate a significant *P* value.

Random effects	Variance		SD	
ID	0.001		0.029	
Batch	0.257		0.507	
Year	0.036		0.191	
	Estimate	SE	*t*	*P*
*Fixed effects*				
Origin (wild-caught)	−2.691e−03	3.745e−02	−0.072	0.943
Season (hot)	2.152e−01	2.809e−02	7.661	**2.55e−14** ^***^
Season (monsoon)	1.995e−01	2.600e−02	7.673	**2.23e−14** ^***^
Age class (elderly elephants)	1.628e−01	6.397e−02	2.545	**0.011** ^*^
Age class (weaned calves)	4.084e−02	5.624e−02	0.726	0.468
Age class (adolescents)	1.011e−01	5.658e−02	1.787	0.074
Age class (prime-aged elephants)	1.147e−01	5.955e−02	1.926	0.054
Sex (male)	6.850e−02	2.169e−02	3.158	**0.002** ^**^
Sex ^*^ age class	NS	NS	NS	0.275
Sex ^*^ season	NS	NS	NS	0.299
*Sample sizes*				
Observations = 3163				
Individuals = 173				
*AICc*				
5173.4				

Finally, we tested the effect of FTM concentration variation on the oxidative status of individuals. To do so, we used linear mixed models (package ‘lme4’, Bates *et al.,* 2015), adjusted with the maximum likelihood method, with FTM concentrations, age class, sex and season as fixed factors, and the ID of each individual, year of sample collection and plate number as random factors.

## Results

### FTM concentrations: association with sex, age class, origin and season

**FIGURE 1:**
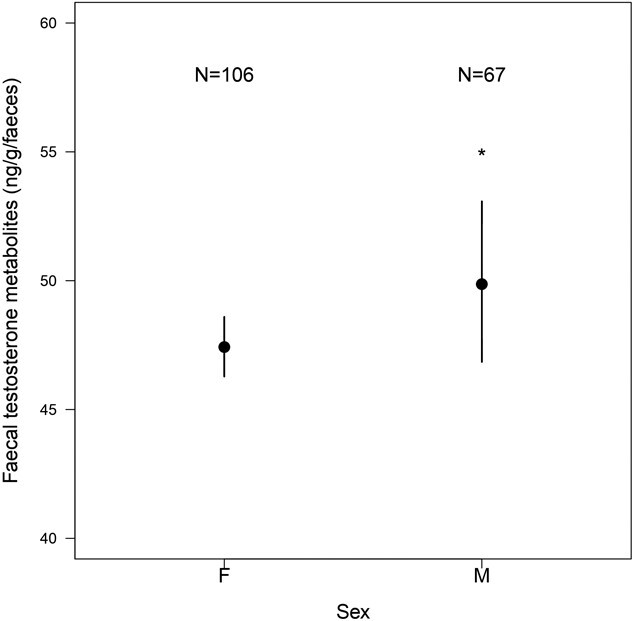
Comparison of FTM concentrations between male and female Asian elephants. The *Y* axis shows the back-transformed FTM concentrations with 95% confidence interval for males and females, corrected for the covariates in the final model in Table 1: season, age class and the origin of the elephants. The asterisk indicates a significant difference between FTM concentrations of males and females, obtained from linear mixed model.

We found significant sex differences in variation in longitudinal FTM concentrations (Table 1), with males having 7.09% higher FTM concentration than females (Fig. 1). FTM concentrations also changed across life stages, increasing with age in both males and females (Table 1, age:sex interaction, P = 0.28). On average, elderly elephants had the highest FTM concentrations (58.3 ± 2.69 ng/g faeces), followed by prime-aged elephants (50.7 ± 1.65 ng/g faeces), adolescents (49 ± 1.31 ng/g faeces), calves-at-heel (48.6 ± 2.63 ng/g faeces) and weaned calves (41.3 ± 0.93 ng/g faeces). Calves-at-heel and weaned calves did not significantly differ in their FTM concentrations (Table 1), whereas the differences between calves-at-heel and adolescent elephants, as well as between calves-at-heel and prime-aged elephants were larger but non-significant (Table 1). FTM concentrations in elderly elephants were significantly higher than FTM concentrations in calves-at-heel (Table 1) and weaned calves (β = 0.122 ± 0.040, t = 3.02, P = 0.003) elephants (Fig. 3). Moreover, both prime-aged elephants (β = −0.074 ± 0.033, t = −2.26, P = 0.026) and adolescents (β = −0.06 ± 0.025, t = −2.39, P = 0.017) had significantly higher FTM concentrations than weaned calves. We did not detect any effect of elephant origin (captive-born or wild-caught) on FTM concentrations (Table 1).

**FIGURE 2:**
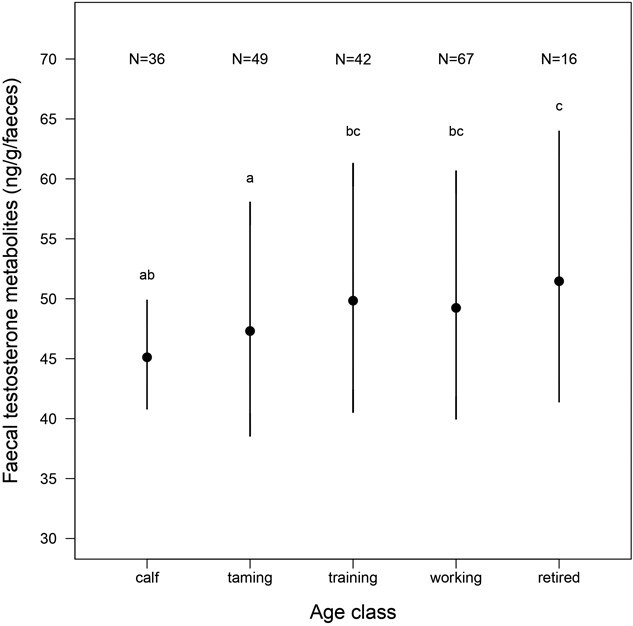
Variation of FTM concentrations through life stages. The *Y* axis shows the back-transformed FTM concentrations with 95% confidence interval as a function of the age classes, corrected for the covariates in the final model in Table 1: sex, season and the status of the elephants. *N* indicates the number of individuals in each category. The results of the post hoc test are represented with letters above the graph. Two different letters for two modalities of the factor indicate that FTM concentrations are significantly different.

In addition, we observed an effect of the environment, with significant seasonal variation in FTM concentrations (Table 1). Whilst elephants showed no significant difference in FTM concentrations between the hot and monsoon seasons (β = 0.016 ± 0.028, t = 0.55, P = 0.58), their FTM concentrations were 24% higher in the hot season and 22% higher in monsoon than in the cold season (Table 1; Fig. 2). This seasonal variation in FTM concentrations was similar for males and females (season:sex interaction, P = 0.30, Table 1).

### FTM concentrations: association with stress markers

We obtained different results for our two indicators of physiological stress: the H/L ratio and the concentration of FGM.

The H/L ratio ranged from 0.21 to 5.6, with an average value of 1.16 ± 0.04. We found no significant link between the H/L ratio and FTM concentrations (Table 3).

By contrast, we found a strong positive relation between FTM concentrations and FGM concentrations (Table 2; Fig. 4). In our data, FGM concentrations ranged from 3.2 to 409.72 ng/g faeces, with an average value of 70.64 ± 0.57 ng/g faeces. For each one-unit increase in FGM, FTM concentrations increased by about 0.85%. Although the positive correlation between FTM concentrations and FGM concentrations seems driven by two extreme values of FGM in Fig. 4, the significance of the correlation remained similar after removing these data points from the analysis (*β* = 0.009 ± 4.1e−4, *t* = 22.25, *P* < 2.2e−16).

**Table 2:** Output of the linear mixed model testing the relation between FGM and FTM concentrations, obtained with the summary of the model. Values in bold indicate a significant *P* value.

Random effects	Variance		SD	
ID	0.000		0.000	
Batch	0.461		0.679	
Year	0.063		0.250	
	Estimate	SE	*t*	*P*
*Fixed effects*				
FGM	8.421e−03	3.861e−04	21.811	**< 2e−16** ^***^
Sex (male)	7.819e−02	2.012e−02	3.887	**1.04e−4** ^***^
Age class (elderly elephants)	1.757e−01	6.053e−02	2.902	**0.004** ^**^
Age class (weaned calves)	7.301e−02	5.472e−02	1.334	0.182
Age class (adolescents)	1.344e−01	5.492e−02	2.447	**0.014** ^*^
Age class (prime-aged elephants)	1.400e−01	5.577e−02	2.511	**0.012** ^*^
Season (hot)	2.204e−01	2.740e−02	8.046	**1.25e−15** ^***^
Season (monsoon)	2.108e−01	2.574e−02	8.190	**3.85e−16** ^***^
*Sample sizes*				
Observations = 2964				
Individuals = 171				
*AICc*				
4486.7				

**Table 3:** Results of the linear mixed model, testing the relation between the H/L ratio and FTM concentrations, obtained with the summary of the model

Random effects	Variance		SD	
ID	0.000		0.000	
Batch	0.451		0.672	
	Estimate	SE	*t*	*P*
*Fixed effects*				
H/L ratio	−0.002	0.047	−0.036	0.971
Sex (male)	0.129	0.064	2.006	**0.046** ^*^
Age class (elderly elephants)	0.191	0.510	0.374	0.709
Age class (weaned calves)	0.106	0.499	0.213	0.832
Age class (adolescents)	0.322	0.501	0.642	0.521
Age class (prime-aged elephants)	0.231	0.504	0.458	0.648
Season (hot)	0.497	0.110	4.515	**9.40e**−06^***^
Season (monsoon)	0.249	0.331	0.753	0.453
*Sample sizes*				
Observations = 274				
Individuals = 89				
*AICc*				
436.4				

**FIGURE 3:**
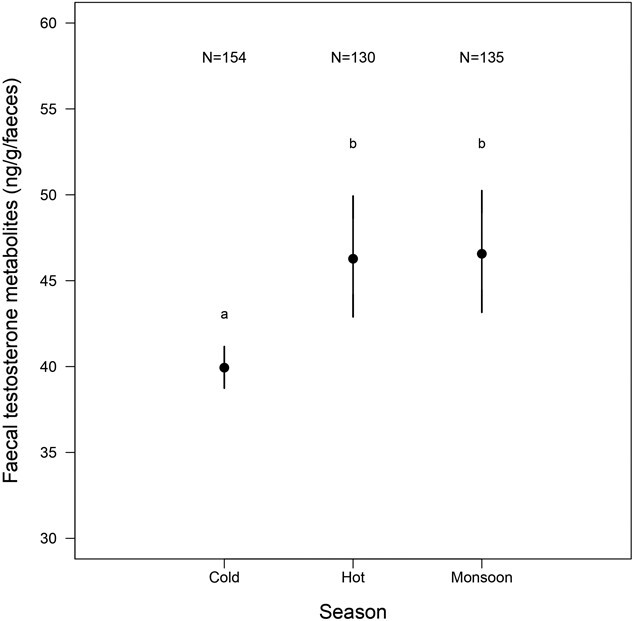
Variation of FTM concentrations through the three seasons in Myanmar. The *Y* axis shows back-transformed FTM concentrations with 95% confidence interval in the three different seasons, corrected for the covariates in the final model in Table 1: sex, age class and status of the elephants. *N* indicates the number of individuals in each category. The results of the *post hoc* test are represented with letters above the graph. Two different letters for two modalities of the factor indicate that FTM concentrations are significantly different.

**FIGURE 4:**
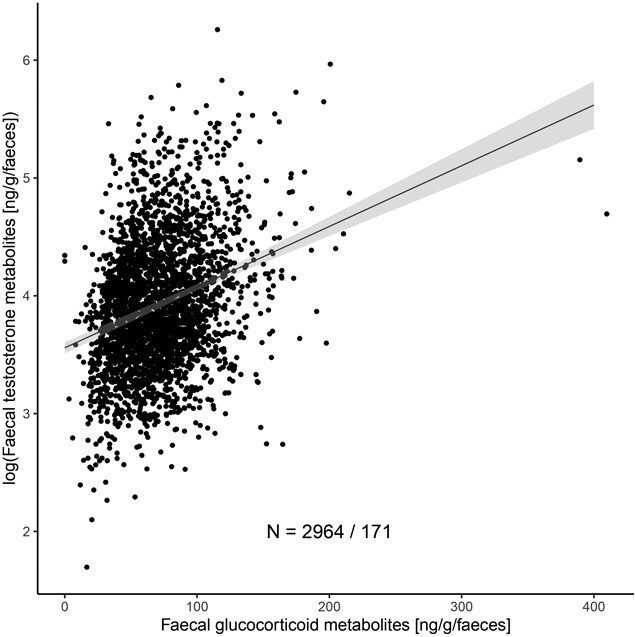
FTM concentrations as a function of FGM concentrations. The *Y* axis shows the log-transformed FTM concentrations with 95% confidence intervals, corrected for the covariates season, age class and sex of the elephants as tested in the linear mixed model in Table 2. The line represents the predicted values of FTM concentrations, and *N* is the number of values/total number of individuals. Note that the association was even more pronounced when repeating the analysis without the two highest FGM values.

### FTM concentrations: association with oxidative status

Finally, we found no significant relationship between FTM concentrations and the concentration of ROMs (Table 4), products of oxidative stress or between FTM concentrations and the activity of SOD (Table 5), an enzyme with antioxidant activity.

## Discussion

Our results indicate that FTM concentration in the Asian elephant is a highly variable trait that responds to external and internal changes. We found that the concentrations of this androgen hormone differed between males and females, across different life stages and between seasons (which are known to affect health and mortality risks in the population) (Mumby *et al.,* 2013a, 2013b; dos Santos *et al.,* 2020). Specifically, FTM concentrations were overall higher in males than females, increased with life stages and were higher during monsoon and hot seasons compared to the cold season. Although we did not detect any link between H/L ratio and FTM concentrations, we found a strong positive correlation between FTM and FGM concentrations. Finally, we found no significant relation between FTM concentrations and the oxidative status of the individuals (i.e. ROM and SOD).

As expected, we observed significantly higher FTM concentrations in males than females, confirming the results of another study on Asian elephants (Rasmussen *et al.,* 1984). This result is consistent with general observations in birds (Møller *et al.,* 2005) and mammals, such as spotted hyenas (Glickman *et al.,* 1987, 1992; Goymann *et al.,* 2001), California ground squirrels (Holekamp and Talamantes, 1991), European moles (Whitworth *et al.,* 1999), bonobos (Dittami *et al.,* 2008) and dugongs (Burgess *et al.,* 2012). However, the difference between sexes was not striking in our results, with males having 7.09% higher mean FTM concentrations than females (Fig. 1), likely due to the absence of males in musth in our data. In this study, we investigated the basal FTM levels in males and females. Males were sampled outside the musth, a periodic state in mature Asian and African bull elephants marked by elevated testosterone levels in blood, urine and faeces (Poole *et al.,* 1984; Rasmussen *et al.,* 1996; Sukumar, 2003; Brown *et al.,* 2007; Kumar *et al.,* 2014), associated with higher aggressiveness and temporal gland secretion (Rasmussen *et al.,* 1984; Poole, 1987; Sukumar, 2003; Ganswindt *et al.,* 2005). Our study indicates that outside of musth, FTM concentrations are surprisingly similar between the sexes. Unlike many other mammals living in seasonal environments, Asian elephants do not breed at a specific time of the year. Males can reproduce all year round and females have 14–16 weeks of reproductive cycle with an ovulation occurring in any season (Fowler and Mikota, 2006; Hildebrandt *et al.,* 2011). This reproductive flexibility could explain the similar FTM concentrations between males and females. Additionally, social factors such as mating and competition might have influenced FTM concentrations, as it influences testosterone secretion in birds (Wingfield *et al.,* 1990) and mammals (Taylor *et al.,* 1989; McDonnell and Murray, 1995; Christensen *et al.,* 2009), including Asian elephants (LaDue *et al.,* 2023). We could not control for these variables in our study, but investigating their effects on androgen hormones could provide further insights.

**Table 4:** Results of the linear mixed model, testing the effect of FTM concentrations on ROM, obtained with the summary of the model

Random effects	Variance		SD	
ID	1.526		1.235	
Plate	9.169		3.028	
Year	15.955		3.994	
	Estimate	SE	*t*	*P*
*Fixed effects*				
FTM	0.003455	0.005808	0.595	0.55230
Sex (male)	1.045526	0.569493	1.836	0.07105
Age class (elderly elephants)	−0.035206	2.852814	−0.012	0.99016
Age class (weaned calves)	0.413090	2.524311	0.164	0.87011
Age class (adolescents)	0.470738	2.606332	0.181	0.85678
Age class (prime-aged elephants)	−0.179146	2.680152	−0.067	0.94675
Season (hot)	−2.624301	0.647511	−4.053	**6.30e−05** ^***^
Season (monsoon)	−1.530216	0.571115	−2.679	**0.00779** ^**^
*Sample sizes*				
Observations = 356				
Individuals = 81				
*AICc*				
2099.7				

**TABLE 5:** Results of the linear mixed model, testing the effect of FTM concentrations on SOD activity, obtained with the summary of the model

Random effects	Variance		SD	
ID	0.036		0.190	
Plate	0.142		0.377	
Year	0.010		0.098	
	Estimate	SE	*t*	*P*
*Fixed effects*				
FTM	2.029e−04	5.616e−04	0.361	0.718
Sex (male)	−3.725e−02	6.623e−02	−0.562	0.576
Age class (elderly elephants)	5.864e−01	3.173e−01	1.848	0.065
Age class (weaned calves)	4.667e−01	2.841e−01	1.643	0.101
Age class (adolescents)	4.891e−01	2.934e−01	1.667	0.096
Age class (prime-aged elephants)	5.245e−01	3.007e−01	1.744	0.082
Season (hot)	−4.797e−02	6.322e−02	−0.759	0.449
Season (monsoon)	−8.734e−02	5.602e−02	−1.559	0.120
*Sample sizes*				
Observations = 438				
Individuals = 91				
*AICc*				
647.4				

Regarding the variation with age, we found an overall trend of increasing FTM concentrations across life stages in both sexes, with the highest concentrations observed in elderly elephants (Fig. 3). FTM concentrations were higher in adolescents, prime-aged elephants and elderly elephants compared to calves-at-heel and weaned calves, which are not yet sexually mature. Sexual maturity typically occurs at 14–15 years in males and females but can occur as early as 8–9 years (Shoshani and Eisenberg, 1982). When comparing FTM concentrations between immature (<14 years old) and mature individuals (see Table A5 in appendix), mature individuals showed significantly higher FTM levels, consistent with the findings of Brown *et al.* (2007), who reported a marked increase in testosterone levels in Asian elephants around 20 years of age. The surprisingly elevated FTM concentrations in calves-at-heel, which did not significantly differ from weaned calves, adolescents and prime-aged elephants, may result from hormone transfer during lactation. Hormonal transfer through mothers’ milk has been demonstrated for glucocorticoids (Brummelte *et al.,* 2010), which can influence offspring growth and behaviour, but such maternal transfer needs to be investigated for testosterone and in Asian elephants. Contrary to our hypothesis, elderly elephants (i.e. > 55 years old) exhibited the highest FTM concentrations. We did not observe a decrease in testosterone concentrations in older individuals, as reported by Thongtip *et al.* (2008) and Glaeser *et al.* (2022) in captive male Asian elephants. A potential explanation is the presence of a selective disappearance effect, where only high-quality individuals with higher FTM concentrations and longer lifespans remain in the oldest age class. Another explanation could be increased sexual activity at older ages. In both Asian and African elephants, males’ social rank, which is size dependent, increases as they continue growing in size and weight with age (Poole, 1989; Lindeque and Jaarsveld, 1993; Lee and Moss, 1995; Sukumar, 2003; Chapman *et al.,* 2016; Lalande *et al.,* 2022). Larger, older African bulls are preferred sexual partners by females and have higher reproductive success (Sukumar, 2003). Moreover, while previous studies have focused primarily on males, our findings were consistent between males and females, with no significant interaction between sex and age class. In female Asian elephants, testosterone levels positively correlate with progesterone concentrations during the oestrous cycle and increase during the luteal phase (Taya *et al.,* 1991). This suggests the production of testosterone by the ovaries in this genus (Taya *et al.,* 1991), a phenomenon well-known in humans (e.g. Kirschner and Wayne Bardin, 1972; Burger, 2002). Hence, testosterone levels may also rise in females as they reach sexual maturity. Furthermore, Asian elephants remain sexually active and can reproduce into advanced ages, with reproduction beyond 60 years reported in females (Sukumar, 2003; Lahdenperä *et al.,* 2014). The higher sexual activity of older elephants may explain the observed increase in FTM concentrations, but further research is needed, especially in females, to better understand this pattern.

As expected, we evidenced strong seasonal variation in FTM concentrations. The climate in Myanmar is defined by three distinct seasons. The hot season is characterized by low humidity, high temperatures and low food availability. In contrast, monsoon is a rainy season with significant rainfall, high temperatures, higher food availability and easier access to water. Finally, the cold season is marked by low temperatures, lower precipitation and better food availability than the hot season, especially at the beginning of the season. Previous studies on MTE elephants found that these seasonal changes influence the elephants’ health, survival and stress markers (FGM and H/L ratio) (Mumby *et al.,* 2013a; dos Santos *et al.,* 2020; Ukonaho *et al.,* 2023). Specifically, stress markers were higher during the cold season (Ukonaho *et al.,* 2023) and survival rates were higher during monsoon (Mumby *et al.,* 2013a). In this study, we observed higher FTM concentrations during monsoon and the hot season than the cold season for both sexes. Glucocorticoids might have suppressed testosterone secretion by inhibiting the HPG axis during the cold season. However, we cannot confirm this hypothesis here, as we found a positive correlation between biomarkers of stress and FTM concentrations. In Thailand, captive male Asian elephants also exhibit higher testosterone concentrations during monsoon (Thongtip *et al.,* 2008; Norkaew *et al.,* 2019). However, contrary to our results, they show higher testosterone concentrations in the cold than in the hot season. The seasonal variation in FTM concentrations is likely influenced by fluctuations in food availability, as suggested by Cooper *et al.* (1990). Testosterone secretion is tightly linked to diet, food intake and nutritional status in male vertebrates (Wilson *et al.,* 1979; Wingfield, 1988; Pérez-Rodríguez *et al.,* 2006; Ruiz *et al.,* 2010). Monsoon is an energetically favourable season with better food availability, providing elephants with a higher-quality diet, which could explain the higher FTM concentrations observed during this season (Thongtip *et al.,* 2008; Norkaew *et al.,* 2019). In addition, when male captive Asian elephants are fed *ad libitum*, FTM concentration correlates positively with temperature and humidity (de Andrés *et al.,* 2021), suggesting that these seasonal factors also affect testosterone secretion. For female captive Asian elephants, body condition is better during monsoon, the luteal phase and oestrous cycle are longer and peaks of progesterone levels are higher (Yang *et al.,* 2023), which correlates positively with testosterone concentrations (Taya *et al.,* 1991). Thus, the seasonal variation in food availability may influence body condition in elephants and thereby affecting testosterone secretion by the testes in males and the ovaries in females. Finally, the higher FTM concentrations observed during both monsoon and the hot season might be related to the reproductive activity of the elephants. Musth in male Asian elephants is more observed during monsoon (Kahl and Armstrong, 2002), and a peak of conceptions in MTE elephants occurs during the hot season (Mumby *et al.,* 2013b, 2015), reflecting a higher sexual activity during these seasons. Hence, FTM concentrations in elephants are influenced by seasonal environmental variations, which might be underpinned by fluctuating food availability and increased sexual activity.

Our results on the relationship between stress measures and FTM concentrations are contradictory. We found no association between FTM concentrations and the H/L ratio. Although this result differs from our predictions, a previous study on the house sparrow found that an experimental elevation of testosterone concentrations with testosterone implants did not change the H/L ratio (Buchanan *et al.,* 2003), suggesting an absence of association between testosterone secretion and stress response. This analysis was performed on a smaller sample size, which may have also reduced statistical power in our study. However, we found a particularly strong relationship between FTM and FGM concentrations using a larger sample size. While stress and glucocorticoids are typically thought to suppress the HPG axis and inhibit reproductive functions (e.g. Saketos *et al.,* 1993; Wingfield and Sapolsky, 2003; Narayan and Parisella, 2017), the positive correlation observed here between androgen and adrenal hormones aligns with findings from other studies on boar (Liptrap and Raeside, 1975, 1978; Borg *et al.,* 1991), pygmy goats (Howland *et al.,* 1985), bison bulls (Mooring *et al.,* 2006) and Asian elephant bulls during musth (Rasmussen *et al.,* 1984; Brown *et al.,* 2007; Yon *et al.,* 2007b, 2007a, 2008; Kumar *et al.,* 2014) (but see de Andrés *et al.,* 2021). The correlation between FGM and FTM may be explained by the release of androgens from the Zona reticularis of the adrenal gland by stimulation of the HPA axis. In addition to glucocorticoids, the HPA axis is involved in the production of adrenal androgens such as dehydroepiandrosterone (Antoniou-Tsigkos *et al.,* 2019; Dutt *et al.,* 2023), which can then be converted into other androgens, including testosterone. Thus, some testosterone may be produced alongside glucocorticoids, potentially explaining the positive correlation observed here. This correlation might also be due to indirect processes. Organisms can develop mechanisms to maintain reproduction under stress, reviewed in Wingfield and Sapolsky (2003). The reproductive system could be resistant to glucocorticoids, especially with chronic stress, and/or the stimulation of the HPG axis, and testosterone secretion could increase to compensate for the inhibitory effects of glucocorticoids to enable reproduction under stress (Place and Kenagy, 2000; Lidgard *et al.,* 2008). Alternatively, stress can occur with reproduction. An elevation of glucocorticoid concentrations is observed during the breeding period in amphibians, reptiles (Moore and Jessop, 2003), Australian marsupials (Bradley *et al.,* 1980; McDonald *et al.,* 1981) and sexual maturation in the Pacific Sockeye salmon (Donaldson and Fagerlund, 1968, 1970). Similarly, sexual signalling, courtships, vocalization and aggressive behaviours require high energetic expenditure, leading to increased corticosterone secretion (Moore and Jessop, 2003). In this study, we lack behavioural data to parallel the physiological markers of stress (Seltmann *et al.,* 2020). Further investigations including behavioural observations are needed to better understand the association between markers of stress and FTM concentrations. Finally, both FGM and FTM concentrations reflect hormonal levels in elephants over approximately 24 hours, while the H/L ratio, influenced by blood cell turnover (30–40 days), captures stress on a different timescale. The contradictory results we observed between these markers of stress may be explained by the differing temporal nature of these physiological measurements.

Regarding the indicators of oxidative stress and the molecular consequences of variations in FTM concentrations, we found no significant relation between FTM concentrations and either ROMs or antioxidant enzyme activity (SOD). The CV from these oxidative stress measurements suggests there is some noise in the measurements, which might explain this absence of a relation. Our results align with Isaksson *et al.* (2011), who reported similar findings in a natural population of White’s skinks. Nonetheless, in the literature, studies examining the relation between testosterone and oxidative stress have produced conflicting results. While elevated testosterone concentrations have been shown to increase oxidative stress, potentially leading to important fitness costs (Chainy *et al.,* 1997; Zhu *et al.,* 1997; Pansarasa *et al.,* 2002; Aydilek *et al.,* 2004; Alonso-Alvarez *et al.,* 2007), other studies suggest that testosterone may have antioxidant properties (Ahlbom *et al.,* 2001; Tam *et al.,* 2003; Guzmán *et al.,* 2005). Hence, we still know little about the effect of testosterone variation on the oxidative status of individuals, and further research is needed to clarify this relation. Finally, a recent study on the same elephant population in Myanmar found no seasonal variation in ROM and SOD levels (Ukonaho *et al.,* 2023). While we observed a significant effect of season on ROM in our results (Table 4), this likely reflects an artefact of the reduced sample size when oxidative stress markers were merged with FTM data. Thus, oxidative stress levels in these elephants appear stable despite seasonal changes and fluctuations in FTM concentrations.

To conclude, our results confirm that FTM concentrations fluctuate with age, sex, seasonal variation and stress levels. Understanding both the intrinsic and extrinsic factors that influence FTM concentration is crucial for comprehending how this hormone shapes individuals’ traits in a changing environment, leading to a diversity of endocrinological phenotypes within and across populations. Listed as endangered on the IUCN red list (Williams et al., 2019), the Asian elephant population is in decline, largely due to habitat loss, fragmentation and vital rates that do not support population growth. A quarter of the remaining Asian elephants are now in captivity, yet these populations also struggle with low birth rates and high infant mortality (Jackson *et al.,* 2019). By identifying the factors influencing FTM concentrations, our study offers critical insights to enhance conservation efforts for this endangered species. As an androgen hormone, testosterone plays a key role in regulating reproductive physiology and behaviour, directly affecting individual fitness. Knowledge of its determinants is therefore essential for optimizing fertility and reproductive success in captive populations. Additionally, understanding FTM variation in semi-captive populations provides important insights into the reproductive health of wild populations and their adaptation to environmental changes. Ultimately, this knowledge is essential for Asian elephants’ conservation, to improve environmental management and implement effective conservation strategies for this endangered species.

## Supplementary Material

Web_Material_coae076

## Data Availability

Data available on request.
