# Supplementary material for: Testosterone variation in a semi-captive population of Asian elephants in Myanmar
Source: Conserv Physiol. 2024 Nov 22;12(1):coae076. doi: 10.1093/conphys/coae076 (PMC11584279; doi:10.1093/conphys/coae076)

Supplementary materials to the following article

Testosterone variation in a semi-captive population of Asian elephants in Myanmar

Héloïse Moullec[1], Vérane Berger[1], Diogo J. Santos[1], Susanna Ukonaho[1], Lisa Yon[2], Michael Briga[1], U. Kyaw Nyein[3], Virpi Lummaa[1]*, Sophie Reichert[1]*

*shared last authors

[1]Department of Biology, University of Turku, FIN-20014 Turku, Finland

[2]School of Veterinary Medicine & Science, University of Nottingham, Sutton Bonington, LE12 5RD, UK

[3]Myanma Timber Enterprise, MONREC, 11011 Insein Township, Yangon, Myanmar

Table A1. Sample sizes of the number of FTM measurements associated to each factors, and number of individuals. Since blood samples were collected less frequently, the sample sizes vary between measurements of FTM, FGM concentrations, H/L ratio and oxidative status. Faecal samples were collected monthly but blood samples only 3 times per year, and we could not collect blood samples from untamed babies. FTM: faecal testosterone metabolite. FGM: faecal glucocorticoid metabolite. H/L ratio: heterophils and lymphocytes ratio. ROM: reactive oxygen metabolites. SOD: superoxide dismutase.

Explanatory variable	Categories	Number of measurements	Number of individuals
**FTM**	**Total**	**3163**	**173**
Sex	Female	1859	106
	Male	1304	67
Age	Calves	111	36
	Taming	1257	49
	Training	843	42
	Working	653	67
	Retired	299	16
Origin	Wild	376	27
	Captive	2787	146
Season	Cold	1207	154
	Hot	980	130
	Monsoon	976	135
**FGM**		**2964**	**171**
**H/L RATIO**		**274**	**89**
**SOD**		**438**	**91**
**ROM**		**356**	**81**

Table A2. Summary table of raw data of FTM concentrations detailed for each variable, FGM concentrations, H/L ratio, ROM (reactive oxygen species) and SOD (superoxide dismutase). FTM: faecal testosterone metabolite. SD: standard deviation. SE: standard error.

	**FTM (ng/g/faeces)**		
	**Mean**	**SD**	**SE**
**Sex**			
Females	47.8	38.9	0.903
Males	46.2	36.6	1.01
**Age classes**			
Calves-at-heel	48.6	27.8	2.63
Weaned calves	41.3	33	0.93
Adolescents	49	38	1.31
Prime-aged elephants	50.7	42.1	1.65
Elderly elephants	58.3	46.5	2.69
**Season**			
Cold	39.9	33.2	0.955
Hot	50.8	44.2	1.41
Monsoon	52.4	35.3	1.13
**Origin**			
Captive-born	45.8	36.5	0.691
Wild-caught	57.4	46.5	2.4

Table A3. Table of the results from reptR function indicating the individual repeatability (R) with standard error (SE) and confidence interval (CI).

Repeatability for ID
N bootstrap = 1000<br>N permutation = 1000
R = 0.002<br>SE = 0.002<br>CI = [0, 0.009]

Table A4. Output of the linear mixed model, testing the effect of the origin of the elephants (captive-born or wild-caught), sex, maturity (immature elephants, ≤14 years old vs mature elephants, ≥15 years old), and effect of the season on FTM concentrations, obtained with a Type III analysis of variance for the fixed factors and the summary of the model for the random factors. The non-significance of an interaction is indicated by 'N.S.' in the table, and the model was run again without this interaction. FTM: faecal testosterone metabolite.

Fixed effects	*F*	*P*
Origin	$F_{1, 156.07} = 0.005$	0.946
Sex	$F_{1, 82.98} = 8.95$	**0.004**

Maturity	$F_{1, 152.02} = 8.46$	**0.004**
Season	$F_{2, 2939.63} = 40.21$	**<2.2e-16**
Sex*Maturity	N.S.	N.S.
Random effects	*Variance*	*Std.Dev*
ID	0.001	0.032
Batch	0.256	0.506
Year	0.035	0.188

Observations = 3163
N = 173

Table A5. Number of individuals in each age class according to their origin.

Origin	Calves-at-heel	Weaned calves	Adolescents	Prime-aged elephants	Elderly elephants
Captive-born	36	49	42	43	8
Wild-caught	0	0	0	24	8

Table A6. Output of the function 'dredge' of 'MuMIN' package used to determine the "best model" based on the second order Akaike Information Criterion (AICc).

**(Intercept)**	**Age class**	**Origin**	**Season**	**sex**	**df**	**logLik**	**AICc**	**delta**	**weight**
3.536387458	+		+	+	12	-2573.65	5171.407	0	0.697429
3.536304059	+	+	+	+	13	-2573.65	5173.418	2.011423	0.255109
3.567601807	+		+		11	-2578.51	5179.104	7.697926	0.014857
3.625139549			+	+	8	-2581.74	5179.534	8.127079	0.011987
3.618513717		+	+	+	9	-2580.89	5179.838	8.431355	0.010296
3.567613503	+	+	+		12	-2578.51	5181.119	9.712882	0.005425
3.644700669			+		7	-2584.12	5182.267	10.86012	0.003057
3.640526416		+	+		8	-2583.62	5183.281	11.87422	0.001841
3.598332711	+			+	10	-2612.77	5245.615	74.20818	5.36E-17
3.598286064	+	+		+	11	-2612.77	5247.627	76.22056	1.96E-17
3.629543862	+				9	-2617.65	5253.351	81.94415	1.12E-18
3.629573038	+	+			10	-2617.65	5255.362	83.95497	4.1E-19
3.705637169		+		+	7	-2622.02	5258.078	86.67156	1.05E-19
3.714232813				+	6	-2623.24	5258.5	87.09324	8.54E-20
3.732174093					5	-2625.25	5260.517	89.11081	3.11E-20
3.726346968		+			6	-2624.45	5260.922	89.51575	2.54E-20

Table A7. Parallelism and recovery of testosterone and corticosterone assays.

Testosterone Parallelism	y = -152.86X+79.211	$R^2 = 0.978$
Testosterone Recovery	y = 0.8228X+0.0263	$R^2 = 0.998$
Corticosterone Parallelism	y = -35.432X+90.523	$R^2 = 0.989$
Corticosterone Recovery	y = 1.1696X+0.1503	$R^2 = 0.994$

Figure A1. Residuals of log-transformed FTM concentrations as a function of FGM concentrations, H/L ratio and residuals of ROM and log-transformed SOD as a function of FTM concentrations. The residuals are corrected for the covariates season, age class and the sex of the elephants as tested in the linear mixed model in Tables 2, 3, 4 and 5. The line represents the predicted values of FTM concentrations, ROM and SOD, and N is the number of values / total number of individuals. FTM: faecal testosterone metabolite. FGM: faecal glucocorticoid metabolite. H/L ratio: heterophils and lymphocytes ratio. ROM: reactive oxygen metabolites. SOD: superoxide dismutase.

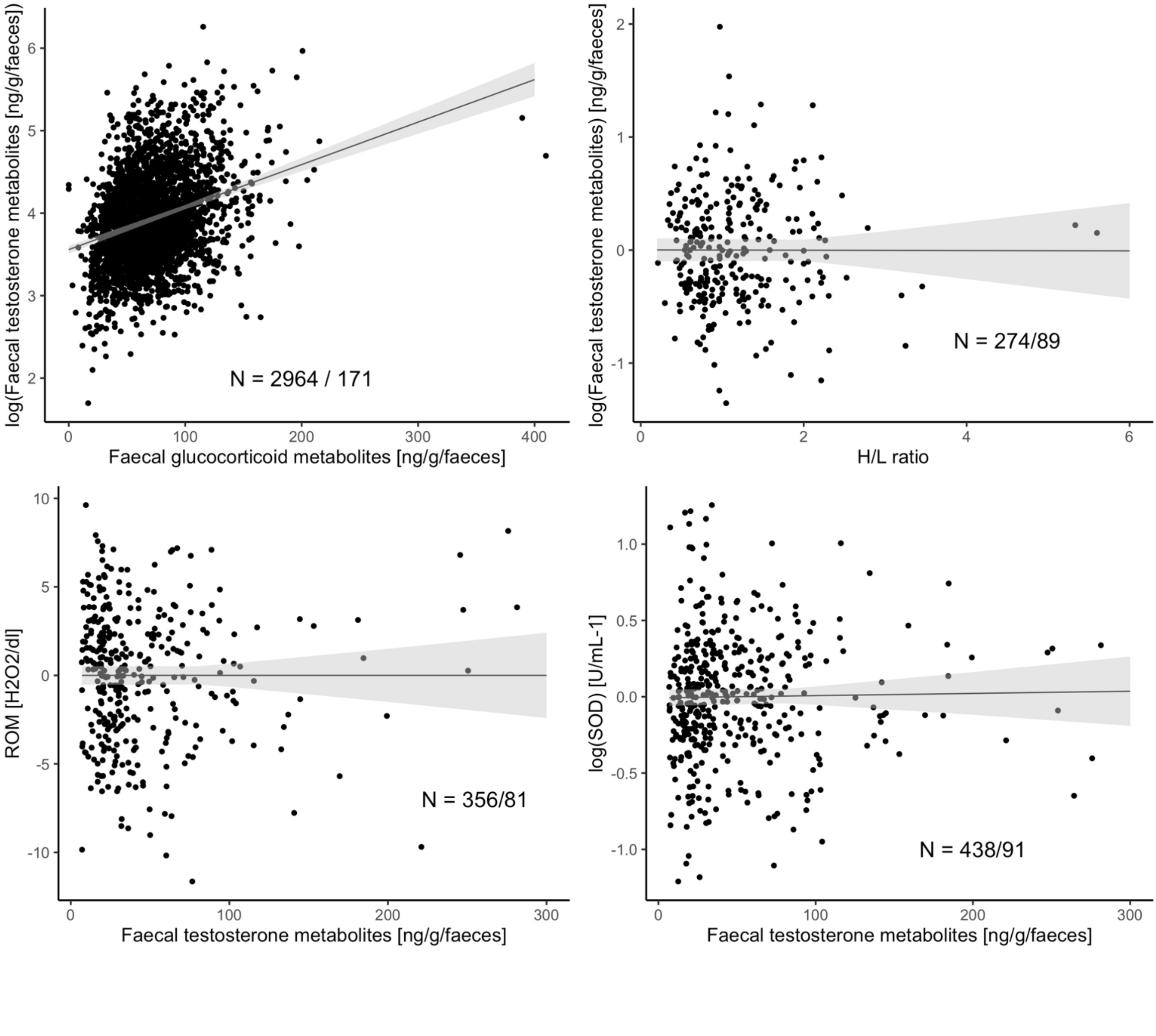

Supplement: Web_Material_coae076 [file web_material_coae076.pdf]
